# Lower serum bilirubin is associated with poor renal outcome in IgA nephropathy patients

**DOI:** 10.7150/ijms.60111

**Published:** 2021-06-11

**Authors:** Zheng Jiang, Jiaxing Tan, Siqing Wang, Lingqiu Dong, Xin Han, Yi Tang, Wei Qin

**Affiliations:** 1 West China School of Medicine, Sichuan University; 2 Division of Nephrology, Department of Medicine, West China Hospital of Sichuan University

**Keywords:** end-stage kidney disease, IgA nephropathy, serum bilirubin, propensity score matching.

## Abstract

**Aims**: IgA nephropathy (IgAN) is the most prevalent primary glomerulonephritis worldwide. We conducted this study to explore the relationship between serum bilirubin and renal outcome of patients with IgAN.

**Methods**: A total of 1492 biopsy proven IgAN patients were recruited and divided into two groups according to their median serum bilirubin concentration: the low bilirubin group (serum bilirubin≤9.7umol/L, n=753) and high bilirubin group (serum bilirubin>9.7umol/L, n=739). Basic clinical characteristics were assessed at the time of renal biopsy and the relationships between serum bilirubin and the combined endpoints were analyzed. The combined endpoints were defined as a 50% decline in estimate glomerular filtration rate (e-GFR), end-stage kidney disease (ESKD), renal transplantation and/or death. In addition, propensity score matching (PSM) was then performed to improve balance and simulate randomization between patients in different groups. Kaplan-Meier survival analysis was applied to explore the role of serum bilirubin in the progression of IgAN. Three clinicopathological models of multivariate Cox regression analysis were established to evaluate the association of serum bilirubin and renal prognosis of IgAN.

**Results:** During median 5-year follow-up period, significant differences were shown in Kaplan-Meier analysis. In the unmatched group, 189 (12.7%) patients progressed to the renal combined endpoints.

Among this, 122 in 753 patients (16.2%) were in low bilirubin group and 67 in 739 patients (9.1%) were in high bilirubin group (p<0.001). After PSM, there were 134 (11.8%) patients reached the combined endpoints, which included 77 in 566 patients (14.6%) in low bilirubin group and 57 in 566 patients (10.1%) in high bilirubin group (p=0.039). The results of three models (including demographics, pathological, clinical indicators and serum bilirubin) demonstrated that a lower basic serum bilirubin level was significantly associated with a higher risk of reaching combined endpoints in IgAN patients both in unmatched and matched cohort.

**Conclusion**: Serum bilirubin level may be negatively associated with the progression of IgAN.

## Introduction

Immunoglobin A nephropathy (IgAN) is featured by IgA diffusely depositing in the kidneys, which is the most prevalent primary glomerulonephritis worldwide. Nearly 15-40% of IgAN patients will progress to end stage renal disease (ESRD) within 20 years 1 .It is quite meaningful to identify risk factors related to the progression of IgAN. Early intervention of these factors may delay the development of IgAN. It is reported that the pathogenesis of IgAN is associated with oxidative stress 2-4. The dynamic balance between oxidation and antioxidation has been destroyed in patients with IgAN 4. It was reported that intrarenal immunoreactivity of heme oxygenase-1(HO-1) was increased in IgAN 5, which can degrade heme to produce carbon monoxide, iron and biliverdin (BV), which is reduced to bilirubin by biliverdin reductase 6. Therefore, serum bilirubin is regarded as a powerful endogenous antioxidant. Many studies have clarified the role of serum bilirubin in diabetes mellitus, renal transplantation, stroke, cardiovascular and metabolic disease 7-9. However, there still lack of research on the relationship between serum bilirubin and IgAN. Whether the level of serum bilirubin could be another predictor of progression of IgAN remains unknown. To clarify these issues, we conducted this study.

## Materials and Methods

### Patients

Biopsy-proven IgAN patients from West China hospital of Sichuan university between May 2009 and September 2019 were enrolled in this study. The Inclusion criteria were: (1) patients confirmed as IgAN by renal biopsy; (2) patients were followed up for at least 6 months or reached the endpoint of study; (3) patients ≥14 years old. The exclusion criteria applied were as follow: (1) patients with systemic disease, such as systemic lupus erythematosus (SLE), Henoch-Schönlein purpura (HSP), liver cirrhosis or disorder of liver function, malignancy etc.; (2) patients without complete renal biopsy or clinical data. Patients were followed up in the out-patient department of West China Hospital or by regular phone calls. The study was approved by the Ethical Committee of West China Hospital of Sichuan University. Informed consent was obtained from each patient or legal guardians prior to treatment.

### Data Collection

Electronic medical records were used to obtain patients' information including age, gender, clinical manifestations, laboratory indexes, renal pathology report, systolic and diastolic blood pressure and treatment strategies. Laboratory values included 24h proteinuria (UPRO), hematuria level (URBC), hemoglobin (Hb), serum total bilirubin (TB), conjugated bilirubin (CB), unconjugated bilirubin (UCB), alanine aminotransferase (ALT), aspartate aminotransferase (AST), serum albumin (ALB), serum creatinine (Scr), estimate glomerular filtration rate (eGFR), uric acid (UA), triglycerides (TG), total cholesterol (TC). The Chronic Kidney Disease Epidemiology Collaboration (CKD-EPI) formula was used to calculate eGFR. Nephrotic syndrome (NS) was defined as 24h proteinuria>3.5g/d and serum albumin<30g/L. Anemia was defined as hemoglobin<110g/L in women and<120g/L in men. The pathological definitions of mesangial proliferation, endocapillary proliferation, segmental glomerulosclerosis, tubular atrophy/interstitial fibrosis, crescents were according to the Oxford classification 10.

### Treatments

Treatment regimens were decided by patients and their doctors according to the clinical and pathological features of the patients. All patients received optimal support treatment including full dose of ACEI/ARB. Glucocorticoids were used when there was persistent proteinuria (>1g/d) after optimal support treatment, 0.5-1mg/kg prednisone daily and tapering down gradually within 6-8 months. Immunosuppressants would be considered in patients with impaired kidney function (Scr>1.5 mg/dl) or rapidly progressive kidney function decline. Immunosuppressants included cyclophosphamide (2mg/kg daily for 3 months), mycophenolate mofetil (1-2g daily for 6-8 months), tacrolimus (0.03-0.05 mg/kg daily for 6-8 months) or cyclosporin (3-5 mg/kg daily for 6-8 months).

### Outcomes

The combined endpoints of renal outcome were eGFR decreased≥50% of the baseline level, end-stage kidney disease (ESKD), renal transplantation and/or death. ESKD was defined as eGFR≤15 mL/min/1.73 m^2^ or maintenance renal replacement treatment.

### Statistical analyses

Participants were divided into two groups according to their median baseline serum bilirubin concentration: the low-bilirubin group (serum bilirubin≤9.7umol/L, n=753) and high-bilirubin group (serum bilirubin>9.7umol/L, n=739). To lessen the selection bias between different groups in this study, propensity score matching (PSM) was performed to adjust for sex, ALT, TG, TC, T, C, eGFR at a 1:1 ratio. PSM was conducted by “Match It” R package and the “nearest neighbor matching” method. A caliper of 0.18 propensity score standard deviation was used to match. Two-tailed p<0.05 was considered statistically significant. Categorical data were described as percentages and analyzed using the Chi-square tests. Continuous variables were expressed as mean ± SD or median (first-third interquartile range) and analyzed with the ANOVA, Student^'^s t test or nonparametric Mann-Whitney U test. Kaplan-Meier survival analysis was performed using the log-rank test. The effects of clinical manifestations and pathological features on renal outcomes were assessed by mutivariate Cox regression analysis. All statistical analysis was performed by using IBM SPSS statistics 26.0 software.

## Results

### Baseline characteristics

**Figure 1:**
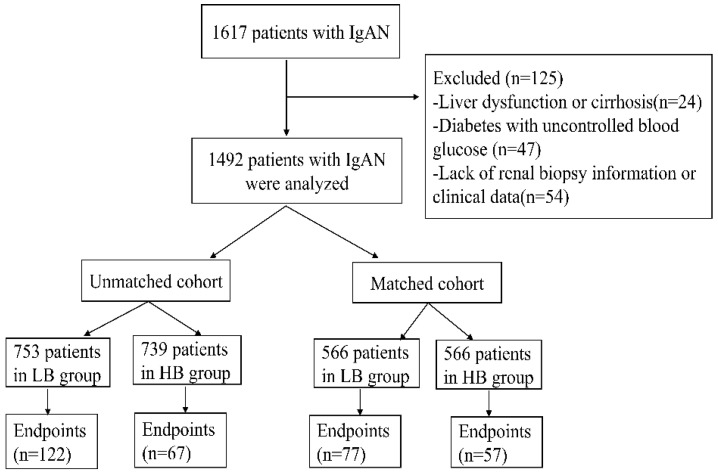
Flow chart of the study.

1617 IgAN patients were recruited in our study. Among this cohort, 125 patients were excluded because of liver dysfunction or cirrhosis (24), diabetes with uncontrolled blood glucose (47) and lack of renal biopsy information or clinical data (54). Finally, 1492 patients were enrolled in this study. The median serum bilirubin of participates was 9.7umol/L (range 2.4-29.7umol/L). And the patients were divided into two groups according to median serum bilirubin concentration: low bilirubin group (LB group: TB ≤9.7umol/L, n=753) and high bilirubin group (HB group: TB>9.7umol/L, n=739) (Figure 1). Patients in LB group had a lower proportion of male (37.5%, p<0.001), lower glucocorticoids (GC) treatment (69.5%, p=0.002), higher nephrotic syndrome (12.2%, p<0.001), severer anemia (16.9%, p<0.001), renal failure (27.9%, p<0.001) and pathological lesions. While patients in HB group tended to have a higher level of UCB(p<0.001), CB(p<0.001), ALT (p=0.001) and lower TG (p<0.001), TC(p<0.001) level. In order to eliminate the difference between two groups, 1132 patients (566 in each group) were enrolled after propensity score matching at a 1:1 ratio. Then there were no differences in clinicopathological manifestations and treatments except serum conjugated and unconjugated bilirubin levels (Table 1).

### Long term renal survival

**Figure 2:**
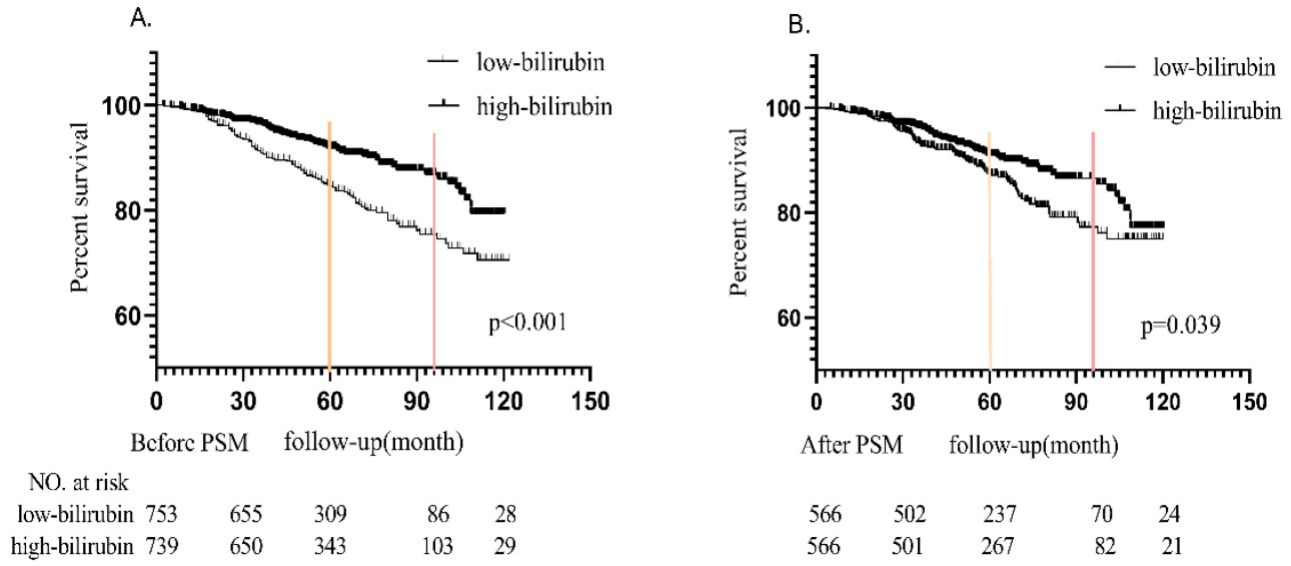
Probability of renal survival between high bilirubin group and low bilirubin group in the unmatched and matched cohort.

The Kaplan-Meier survival curves of the unmatched cohort and matched populations according to the level of serum bilirubin were shown in Figure 2(A and B). During median 5-year follow-up period, in the unmatched cohort, 189 (12.7%) patients progressed to the renal endpoints. It could be noticed that more patients in LB group (122 in 753 patients, 16.2%) reached renal endpoints than those in HB group (67 in 739 patients, 9.1%). In the matched cohort, there were 134 (11.8%) patients reached the combined endpoints, which included 77 in 566 patients (14.6%) in LB group and 57 in 566 patients (10.1%) in HB group. Kaplan-Meier analysis indicated that better renal survival in high bilirubin group compared with those in low bilirubin group in unmatched and matched cohort (p<0.001 and p=0.039, Figure 2). It was found that 5-year renal survival rates were 84.8% (87.5% after PSM) and 91.9% (91% after PSM) in LB and HB groups. 8-year renal survival rates were 75.2% (77.3% after PSM) and 86.9% (86.5% after PSM) in two groups, respectively.

### Factors of renal endpoints

Renal endpoints included eGFR decreased≥50% of the baseline level, end-stage kidney disease (ESKD), renal transplantation and/or death. Multivariate Cox regression model was performed to analysis the relationship between clinicopathological parameters and renal endpoint (Table 2-4). In the unmatched cohort, model 1 (demographics + pathological features + serum total bilirubin), model 2 (demographics + clinical features+ serum total bilirubin) and model 3 (demographics + clinical+ pathological features+ serum total bilirubin) indicated that serum bilirubin was independent factors of renal endpoints (model 1:HR 0.646, 95%CI 0.476-0.877, P=0.005; model 2: HR 0.624, 95%CI 0.451-0.865, P=0.005; model 3:HR 0.651, 95%CI 0.467-0.906, P=0.011). After PSM, the results of model 1(HR 0.705, 95%CI 0.5-0.994, P=0.046), model 2(HR 0.57, 95%CI 0.317-0.819, P=0.002) and model 3(HR 0.568, 95%CI 0.394-0.819, P=0.002) also strongly indicated serum bilirubin as a protective factor of renal prognosis. Based on these results, it could be confirmed that serum bilirubin was an independent protective factor of renal outcomes in IgAN patients.

## Discussion

Although the detailed mechanism of IgAN still remains unknown, the role of oxidative stress in the pathogenesis has attracted more and more attention 2, 11, 12. It was reported that the activation of intracellular signaling in mesangial cells cultured with aberrantly glycosylated IgA and in renal biopsies of IgAN patients basically results in oxidative stress 5. Advanced oxidation protein products (AOPPs) are considered to be important mediators of inflammation 13, 14, which could activate monocytes to release myeloperoxidase and synthesize reactive oxygen species. Increased AOPPs levels were observed in IgAN patients 4, 13, 15. Bilirubin, as a powerful endogenous antioxidant, plays an important role in oxidative stress. It can inhibit NADPH oxidase and further inflammatory reaction 9. However, it is still do not know whether bilirubin has a role in IgAN progression.

In this study, in a cohort of 1492 patients with biopsy-proven IgAN, it was observed that a higher serum bilirubin level was beneficial to the outcome of IgAN. In the current study, it was found that the long-term renal outcome of IgAN patients with higher serum bilirubin level were better than those with lower serum bilirubin level. In the unmatched cohort, 16.2% (122/753) low-bilirubin group patients and 9.1% (67/739) high-bilirubin group patients progressed to the renal endpoints, and the Kaplan-Meier analysis revealed significant difference (p<0.001). The result was consistent in the matched cohort (p=0.039), including 77 (14.6%) patients in low bilirubin group and 57 (10.1%) patients in high bilirubin group reached the endpoints.

In order to simulate randomization and improve balance on baseline variables, propensity score matching analysis was carried out. Multivariate Cox regression analysis was performed with several models consisted of demographics, clinical, pathological indexes and serum bilirubin level, which could contain more confounders to reduce selective bias. The results showed that serum bilirubin level was an independent risk factor in every model in both unmatched and matched cohort. Based on these results, serum bilirubin level could be recognized as a protective factor for long term prognosis of patients with IgAN. Previously, it was reported that serum bilirubin level was associated with the progression of IgAN 16, 17. However, previous studies did not analyze the pathological changes, moreover, selective bias were not adjusted by PSM. Therefore, the results of this study are more reliable than previous studies.

Many studies focused on the relationship between serum bilirubin levels and some oxidative stress diseases including cardiovascular disease, diabetes, obesity 7-9. The antioxidative characteristics of bilirubin might protect not only against the progression of CKD but also against loss of residual kidney function (RKF) in PD patients 18. Elevated bilirubin concentrations were consistently associated with reduced vascular resistance 19, 20, improved eGFR 21, renal tubular function, and slowing of the progression of kidney damage 22. In the animal experiments, the bilirubin-treated mice showed less fibrosis in the unilateral ureteral obstruction (UUO) model and bilirubin treatment decreased fibronectin expression in tubular epithelial cells 23. Besides, it is reported that bilirubin interferes with vascular cell adhesion molecule1 (VCAM-1) and intercellular adhesion molecule1 (ICAM-1) to regulate atherosclerotic lesion formation and vascular inflammation both in animal and human models 9, 24. Furthermore, bilirubin participates in immune response at several levels including modulation of regulatory T cells and T helper type 17 (Th17) cells, inhibition of the Toll-like receptor 4/nuclear factor kappaB signaling pathway, down-regulation of NLRP3 inflammasome 8, 24-26, which has been proved to be very important for IgA nephropathy in a previous study by our team 27.

However, there were still some limitations in our study. First, this was a single center study in China, the patients were mainly Han, Tibetan and Yi Chinese living in the southwest region of China, so it was not known whether our results had distinctive demographic or ethic characters. Second, though we adjusted for potential confounders which were likely to affect the association between serum bilirubin levels and kidney outcomes of IgAN patients, the effect of unadjusted factors such as serum ferritin, haptoglobin and the activity of the hemeoxygenase-1 (HO-1), could not be eliminated in current study. Third, the sample size was limited which may not avoid all bias.

## Conclusion

In conclusion, serum bilirubin level may be negatively associated with the progression of IgAN, which could be applied as a novel predictor in clinic. Future multicenter and perspective studies are needed to confirm these findings and to investigate the underlying mechanisms.

## Figures and Tables

**Table 1:** Baseline characteristics of IgAN patients.

Variables	Before PSM serum bilirubin groups		P value	After PSM serum bilirubin groups		P value
	LB(n=753) ≤9.7mmol/L	HB(n=739) >9.7mmo/L		LB(n=566) ≤9.7mmol/L	HB(n=566) >9.7mmo/L	
Sex (F/M, %)	471/282(62.5/37.5)	350/389(47.4/52.6)	<0.001 **	338/228(59.7/40.3)	332/234(58.7/41.3)	0.762
Age(year)	33(25-42)	32(26-40)	0.135	34.49±11.26	34.45±10.49	0.952
Follow-up (m)	58.55±27.74	61.26±28.08	0.061	59.64±27.77	62.07±27.73	0.141
ALT (IU/L)	18 (13-25)	19 (14-30)	0.001**	18 (13-26)	18 (13-29)	0.058
AST (IU/L)	22.34±8.64	22.88±8.32	0.219	22.16±8.03	22.69±8.5	0.284
TG (mmol/L)	1.62 (1.08-2.4)	1.4 (0.99-1.94)	<0.001**	1.82±1.21	1.82±1.34	0.951
TC (mmol/L)	4.93 (4.17-5.99)	4.7 (3.94-5.42)	<0.001**	4.93±1.28	5±1.31	0.376
URBC (/HP)	22 (7-71.5)	17 (6-52)	0.091	20 (7-65.25)	16 (6-51)	0.055
UA (umol/L)	373.48±107.84	377.01±100.35	0.512	370.38±107.55	373.4±102.33	0.629
LDH(IU/L)	179.28±47.93	176.62±42.56	0.268	173.63±41.2	179.03±51.75	0.056
UCB(umol/L)	5.1(4.1-6.1)	9.4(7.9-11.6)	<0.001**	5.1(4.2-6.1)	9.5(8-11.6)	<0.001**
CB (umol/L)	2(1.5-2.6)	4(3.2-5.1)	<0.001**	2.2(1.7-2.73)	3.85(3-4.8)	<0.001**
ALB(g/L)	38.3(33.9-42.1)	41.2(38.3-44.3)	<0.001**	39.63±5.62	39.58±5.51	0.865
UPRO(g/d)	1.89(0.98-3.16)	1.16(0.69-2.09)	<0.001**	2.06±2.39	1.84±1.8	0.078
NS (N/Y, %)	661/92(87.8/12.2)	724/15(98/2)	<0.001**	551/15(97.3/22.7)	551/15(97.3/22.7)	1
HTN (N/Y, %)	549/204(72.9/27.1)	546/193(73.9/26.1)	0.682	422/144(74.6/25.4)	418/148(73.9/26.1)	0.839
Anemia (N/Y, %)	626/127(83.1/16.9)	690/49(93.4/6.6)	<0.001**	512/54(90.5/9.5)	517/49(91.3/8.7)	0.679
eGFR (%)			<0.001**			0.607
<60ml/min	210 (27.9)	120 (16.2)		121 (21.4)	113 (20)	
>60 ml/min	543 (72.1)	619 (83.8)		445 (78.6)	453 (80)	
M0/M1(%)	169/584 (22.4/77.6)	196/543 (26.5/73.5)	0.071	133/433 (23.5/76.5)	144/422 (25.4/74.6)	0.489
E0/E1(%)	708/45 (94/6)	716/23 (96.9/3.1)	0.009*	539/27 (95.2/4.8)	546/20 (96.5/3.5)	0.372
S0/S1(%)	307/446 (40.8/59.2)	307/432 (41.5/58.5)	0.793	230/336 (40.6/59.4)	219/347 (38.7/61.3)	0.544
T0/T1(%)	559/194 (74.2/25.8)	628/111 (85/15)	<0.001**	449/117 (79.3/20.7)	460/106 (81.3/18.7)	0.455
C0/C1(%)	567/186 (75.3/24.7)	593/146 (80.2/19.8)	0.025*	443/123 (78.3/21.7)	443/123 (78.3/21.7)	1
GC (N/Y)	230/523 (30.5/69.5)	174/565 (23.5/76.5)	0.002*	159/407 (28.1/71.9)	141/425 (24.9/75.1)	0.252

Notes: m month, d day, NS nephrotic syndrome, ALT alanine aminotransferase, AST aspartate aminotransferase, eGFR evaluate glomerular filtration rate, UA uric acid, TG triglycerides, TC total cholesterol, URBC number of erythrocytes in urine at high magnification, LDH lactate dehydrogenase, UCB unconjugated bilirubin, CB conjugated bilirubin, ALB serum albumin, UPRO proteinuria, HTN hypertension, M mesangial hypercellularity, E endocapillary hypercellularity, S segmental, glomerulosclerosis or adhesion, T tubular atrophy/interstitial fibrosis, C crescents, 1stands for presence, 0 stands for absence, N: no, Y: yes. *: p<0.05, **: p<0.001.

**Table 2:** Analysis of factors associated with renal outcomes in model 1 (demographics + pathological features+ serum bilirubin).

Variables	Before PSM			After PSM		
	HR	95% CI	P	HR	95% CI	P
Male/female	1.427	1.064-1.914	0.018*	1.573	1.102-2.245	0.013*
Age (per year)	0.998	0.984-1.012	0.757	1.004	0.988-1.021	0.61
Total bilirubin (High/low)	0.646	0.476-0.877	0.005*	0.705	0.5-0.994	0.046*
M(M1/M0)	1.321	0.737-2.369	0.35	1.156	0.694-1.925	0.578
E(E1/E0)	1.373	0.765-2.464	0.289	1.245	0.548-2.828	0.601
S(S1/S0)	1.877	1.319-2.67	<0.001**	2.198	1.408-3.429	0.001**
T(T1-2/T0)	5.995	4.36-8.244	<0.001**	5.602	3.834-8.186	<0.001**
C(C1-2/C0)	1.008	0.722-1.407	0.964	0.876	0.57-1.346	0.547

Notes: M mesangial hypercellularity, E endocapillary hypercellularity, S segmental glomerulosclerosis or adhesion, T tubular atrophy/interstitial fibrosis, C crescents, *:p<0.05, **:p≤0.001.

**Table 3:** Analysis of factors associated with renal outcomes in model 2 (demographics + clinical indicators+ serum bilirubin).

Variables	Before PSM			After PSM		
	HR	95% CI	P	HR	95% CI	P
Male/female	1.459	1.032-2.063	0.033*	1.502	0.996-2.264	0.052
Age (per year)	0.978	0.963-0.993	0.005*	0.985	0.967-1.004	0.114
Total bilirubin (High/low)	0.624	0.451-0.865	0.005*	0.57	0.317-0.819	0.002*
HTN(N/Y)	0.981	0.696-1.383	0.914	0.857	0.564-1.301	0.469
ALT(IU/L)	0.997	0.982-1.013	0.73	0.999	0.981-1.017	0.898
AST(IU/L)	0.992	0.966-1.018	0.545	0.994	0.963-1.027	0.73
TG (mmol/L)	1.11	0.999-1.232	0.052	1.006	0.866-1.168	0.938
TC (mmol/L)	0.969	0.867-1.084	0.583	1.113	0.976-1.268	0.109
UA (umol/L)	1.003	1.002-1.005	<0.001**	1.004	1.002-1.006	<0.001**
URBC(/HP)	0.998	0.997-1	0.034*	0.998	0.996-1	0.038*
LDH(IU/L)	1.003	1.001-1.006	0.017*	1.003	1-1.007	0.057
CKD stages (stage1-3/4-5)	0.162	0.111-0.237	<0.001**	0.179	0.114-0.28	<0.001**
NS(N/Y)	0.793	0.465-1.352	0.394	1.016	0.445-2.322	0.97
Glucocorticoids (Y/N)	0.861	0.633-1.172	0.342	0.791	0.551-1.137	0.206
Anemia (N/Y)	0.586	0.4-0.858	0.006*	0.621	0.374-1.029	0.065

Notes: NS nephrotic syndrome, ALT alanine aminotransferase, AST aspartate aminotransferase, UA uric acid, TG triglycerides, TC total cholesterol, URBC number of erythrocytes in urine at high magnification, LDH lactate dehydrogenase, HTN hypertension, N: no, Y: yes. *: p<0.05, **: p≤0.001.

**Table 4:** Analysis of factors associated with renal outcomes in model 3 (demographics+ clinical indicators+ pathological features+ serum bilirubin).

Variables	Before PSM			After PSM		
	HR	95% CI	P	HR	95% CI	P
Male/female	1.215	0.863-1.71	0.264	1.397	0.918-2.127	0.118
Age (per year)	0.98	0.964-0.996	0.015*	0.989	0.97-1.008	0.251
Total bilirubin (High/low)	0.651	0.467-0.906	0.011*	0.568	0.394-0.819	0.002*
HTN(N/Y)	0.941	0.661-1.339	0.735	0.919	0.597-1.414	0.7
ALT(IU/L)	0.995	0.979-1.011	0.532	0.995	0.977-1.013	0.568
AST(IU/L)	0.999	0.972-1.026	0.942	1.009	0.976-1.042	0.607
TG (mmol/L)	1.097	0.987-1.219	0.087	1.004	0.857-1.177	0.956
TC (mmol/L)	0.995	0.883-1.122	0.94	1.175	1.023-1.348	0.022*
UA (umol/L)	1.003	1.001-1.004	0.001**	1.003	1.001-1.005	0.002*
URBC(/HP)	0.999	0.997-1	0.08	0.998	0.996-1	0.071
LDH (umol/L)	1.003	1-1.006	0.041*	1.002	0.998-1.005	0.319
CKD stages (stage1-3/4-5)	0.241	0.161-0.36	<0.001**	0.272	0.17-0.436	<0.001**
NS(N/Y)	0.773	0.443-1.348	0.364	1.134	0.483-2.664	0.773
Glucocorticoids (Y/N)	0.974	0.713-1.331	0.869	0.873	0.604-1.262	0.47
Anemia(N/Y)	0.686	0.412-1.141	0.147	0.621	0.371-1.04	0.07
M(M1/M0)	1.13	0.706-1.81	0.611	1.065	0.63-1.8	0.813
E(E1/E0)	1.196	0.647-2.212	0.568	1.229	0.539-2.806	0.624
S(S1/S0)	1.729	1.183-2.528	0.005*	1.882	1.184-2.989	0.007*
T(T1-2/T0)	2.681	1.882-3.819	<0.001**	2.582	1.702-3.916	<0.001**
C(C1-2/C0)	0.992	0.695-1.417	0.965	0.873	0.555-1.372	0.555

Notes: NS nephrotic syndrome, ALT alanine aminotransferase, AST aspartate aminotransferase, UA uric acid, TG triglycerides, TC total cholesterol, URBC number of erythrocytes in urine at high magnification, LDH lactate dehydrogenase, HTN hypertension, M mesangial hypercellularity, E endocapillary hypercellularity, S segmental glomerulosclerosis or adhesion, T tubular atrophy/interstitial fibrosis, C crescents, N: no, Y: yes. *: p<0.05, **: p≤0.001.
